# Effect of amount of biomaterial used for maxillary sinus lift on volume maintenance of grafts

**DOI:** 10.4317/jced.56315

**Published:** 2020-09-01

**Authors:** Luciene-Dornas Mendes, Roberta-Paula-Colen Bustamante, Bruno-César-Ladeira Vidigal, Mario-Nazareno Favato, Flávio-Ricardo Manzi, Mauricio-Greco Cosso, Elton-Gonçalves Zenóbio

**Affiliations:** 1 Post-graduate, Dentistry Department Program Pontifical Catholic University of Minas Gerais, Belo Horizonte, Minas Gerais, Brazil; 2 Post-graduate, Implant Master’s Program Pontifical Catholic University of Minas Gerais, Belo Horizonte, Minas Gerais, Brazil; 3 Associated Professor, Dentistry Department, Implant Master’s Program Pontifical Catholic University of Minas Gerais, Belo Horizonte, Minas Gerais, Brazil

## Abstract

**Background:**

Regardless of the kind of biomaterial used for the graft, it is clear that, over time, the graft undergoes dimensional changes that could influence the final bone volume obtained, which could alter the stability of the installed implants. The aim of the present study was to compared and correlated the graft behavior with the amount (in grams) of xenogeneic and alloplastic biomaterials used in grafts for maxillary sinus lift.

**Material and Methods:**

This retrospective cohort study used 148 CBCT images of 74 grafts from 68 maxillary sinuses lift patients in a university, post-graduate clinic. The weights of biomaterials, categorized in intervals according to amount used, were correlated with the graft volumes at V1 (10 days) and V2 (180 days). Kruskal-Wallis test was used to evaluate the possible bias effect of weight on graft maintenance.

**Results:**

Mean weights of biomaterials used were: Bio-Oss Small® (1.58g); Bio-Oss Large® (1.35g); Endobon® (0.72g); BoneCeramic®+Emdogaim® (0.96g); Cerasorb® (1.13g) and Osteogen® (2.70g). No significant differences (*p*>0.05). Were found for the influence of these mean amounts in graft maintenance: Bio-Oss Small® (18); Bio-Oss Large® (10); Endobon® (17); BoneCeramic®+Emdogaim® (10); Cerasorb® (11); and Osteogen® (08) at V1 and V2. However, when biomaterials were categorized by intervals, all Cerasorb® interval groups showed statistically significant differences (*p*<0.001) in graft volume at V2.

**Conclusions:**

The amounts of the biomaterials used could influence the final volume; depending on the biomaterial characteristics. Implant installation was possible with all studied grafts, although graft volume shrinkage should be considered when selecting biomaterial for sinus lift.

** Key words:**Biocompatible materials; cone beam computed tomography; maxillary sinus; hydroxyapatites.

## Introduction

Biomaterials with osteoconductive capacity were evaluated in maxillary sinus lift, obtaining results equivalent to those of autogenous grafts (1). The xenogeneic and alloplastic hydroxyapatites present as viable alternatives for maxillary sinus lift (2-5).

Regardless of the kind of biomaterial used for the graft, it is clear that, over time, the graft undergoes dimensional changes that could influence the final bone volume obtained, which could alter the stability of the installed implants (2-6).

The use of computed tomography (CT) to analyze the bone volume achieved after maxillary sinus is reported in the literature as an accurate method, which produces reliable images with little distortion (6-14).

In this context, there are studies (4,6,11-22) using different biomaterials evaluating the graft shrinkage and the bone formation. However, scientific information concerning the influence of the amount of biomaterial used to obtain the initial and final volumes of the graft during maxillary sinus lift, has still not been identified in the literature.

There being several alternatives regarding the choice of graft biomaterials for maxillary sinus lift, the present study aims to evaluate the possible bias effect of the amount of biomaterials on graft volume maintenance, comparing and correlating graft volumes with the amount (in grams) of different xenogeneic and alloplastic biomaterials, used for maxillary sinus lift grafts.

## Material and Methods

The present study was approved by the PUCMINAS - Catholic University, Post-graduate Research Implant Clinic, Belo Horizonte, Brazil, - ethics committee, number CAAE 02663212.9.0000.5137.

This retrospective cohort observational study analyzed 80 maxillary sinuses lift patients records from PUCMINAS between 2014 and 2018. After inclusion and exclusion patients’ criteria, the present study used 68 patients records, whose was planning maxillary implant overdenture prosthesis.

-Patient selection

The inclusion criteria patients’ data selection was determined by the complete maxillary tomographic exams obtained between the 5th and 7th day and 180-day post-surgery. It’s important to stress that the tomographic exam in the first period of the study at 7 to 10 days was done as part of the University Surgical Clinical protocol (11-13), to check correct graft accommodation, any infiltration of the biomaterial in the sinus cavity, membrane height or other intercurrence that could interfere with the sinus graft healing or could induced in maxillary sinusitis (23-25) and 180-day post-surgery was done to plan the implant installation. All those patients were maxillary complete edentulous, with a residual alveolar ridge height <4mm, and a total mean volume found for the maxillary sinuses of 15.65cm3 (13).

As exclusion criteria patients had autoimmune diseases, diabetes mellitus, alcoholism, smoker, stress (required sedation), active periodontal diseases, maxillary sinus diseases or surgeries, or that had some intercurrences like membrane perforations, biomaterials sinus incursions, sinus infections, during or after the sinus lift graft procedure, were removed from the data study.

-Surgical procedure

All surgeries were performed by clinical specialists according PUCMINAS Implant Dentistry Department protocol (11,12). After administering local anesthesia with lidocaine 1:100.000 (Alphacaína® - DFL) a horizontal incision was made on the crest of the alveolar ridge and two vertical incisions beyond the gingival mucus line. A full thickness flap exposed the lateral wall of the maxillary sinus. An oval-shaped bone window with a final regular size of 08mm to 10mm height and 10mm to 18mm width, was obtained by osteotomy of the maxillary sinus lateral wall. A number 8 spherical diamond drill was used under constant sterile saline irrigation for this procedure. The remaining bone in the center of the window, attached to the maxillary sinus membrane, was folded into the sinus cavity during the detachment of the membrane.

The sinus cavity obtained was filled with a selected biomaterial according to the previously choice of surgeons and patients. The biomaterials used were: anorganic bovine bone (BioOss®-Small and Bio-Oss® Large, Geistlich Biomaterials, Wolhusen, Switzerland); anorganic bovine (Endobon® RegenerOsst, BIOMET3i, Palm Beach Gardens, FL, USA); biphasic phosphate Bone Ceramic® + protein enamel matrix Emdogain® (Straumann, Basel, Switzerland), tricalcium phosphate (Cerasorb® M Dental Curasan, Frankfurt/Main, Germany); and hydroxyapatite (Osteogen®, Intra-lock® System, USA).

The amount of biomaterial used during the sinus lift surgery is determined according to the number of implants and their spatial distribution based on the prosthetic planning for overdentures using a total of four implants. All the biomaterials were carefully applied dry in the surgical sinus areas, according to the necessary amount. Regarding the intended degree of the height sinus filling, all the grafts must permit the installation of 9mm to 11mm long implants, after a healing period of 180 days. A sample of each biomaterial was weighed using a precision balance to check that it complies with the manufacturer’s information, to a precision of 0.01g. The bone window was covered with a collagen membrane (Surgidry-film, Technodry, Brazil) and the flap was repositioned without tension and sutured appropriately by first intention using 0.5 Nylon (Ethicon, Johnson, USA) suture. Medications were prescribed, including: 0.12% chlorhexidine gel twice daily for 15 days in the operated area, 500mg of Amoxicillin every 8 hours for 10 days, Paracetamol 750mg every 6 hours for three days and Benzalkonium Chloride (0.1mg + 9.0 mg Sodium Chloride - Sorine®) nasal solution four drops in each nostril six times per day for 10 days. The sutures were removed 10 days after the surgery and the patients were monitored throughout the entire period.

The biomaterials used to graft the 74 sinuses analyzed were: Bio-Oss® Small=18, Bio-Oss® Large=10, Osteogen®=08, Bone Ceramic® + Emdogain®=10, Cerasorb®=11, Endobon®=17.

-CBCT evaluation

**Figure 1:**
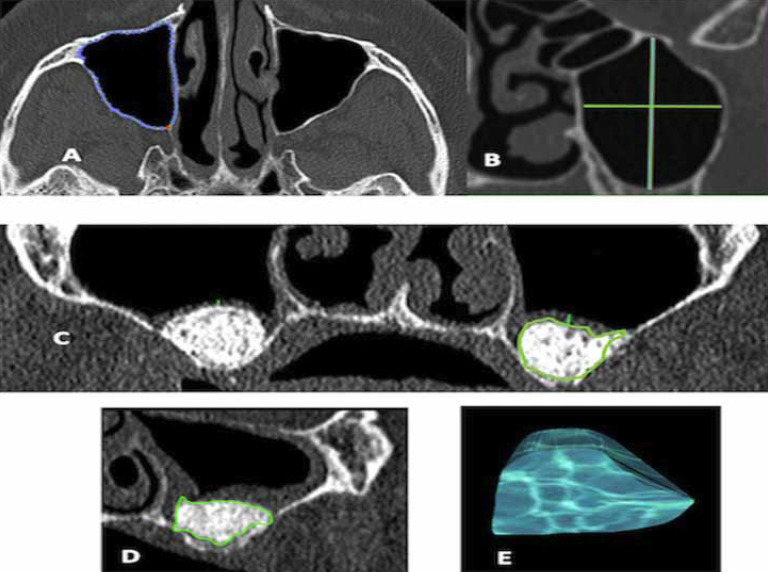
A total sinus volume was calculated after a manually delimited of area in several axial cuts A, and sagittal linear measurement sinus height and width B. A total sinus volume of the graft was calculated after a manually delimited of area in several axial C and sagittal cuts D. The program calculated automatically the volume E.

During the study period, at V1 (7 days) and V2 (180 days), a total of 148 CBCT images, were obtained. The tomography images were analyzed by a single, expert, trained and blinded operator using Osirix® MD Imaging 6.5 software (Pixmeo, Geneva, Switzerland), with a Kappa concordance index of 0.79. The variables maxillary sinus size (total volume), with the mesiodistal, buccal-palatal distances and height, were measured, and correlated with the volumetric changes of the grafts during the two healing periods. The images were delimited manually by the evaluator, and the program semi-automatically defined all sagittal, axial and coronal reconstructions of the images as described for Favato (13), (Fig. 1).

-Statistical analysis

The Kolmogorov-Smirnov test was used to determine the normal distribution of the data. The Kruskal-Wallis test was used at V1 and V2 to analyze the graft volume maintenance and the influence of the amount of biomaterials used.

## Results

The final sample consisted of 148 computed tomography (CBCT) images of 74 grafts from 68 posterior maxillary edentulous patients, 23 males and 45 females, mean age of 56 years, referred for sinus lift.

The amounts of biomaterials used were categorized in the following intervals, show in Table 1. The variable evaluated was the influence of the amount (weight in grams) of different biomaterials used in sinus lift, by measuring the volumetric changes of the grafts at 7 days and at 180 days.

**Table 1:**
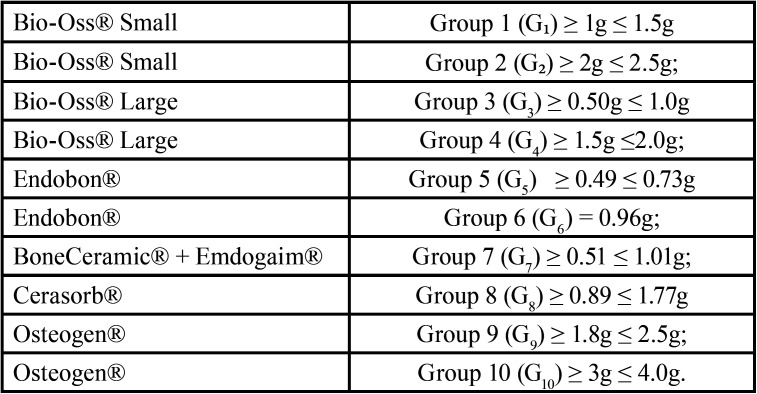
Amounts of biomaterials used categorized in the following intervals.

The mean amounts of biomaterials used were: 1.58g of Bio-Oss® Small, 1.35g of Bio-Oss® Large, 0.72g of Endobon®, 0.96g of BoneCeramic® + Emdogaim®, 1.13g of Cerasorb® and 2.70g of Osteogen®. The means, medians and standard deviations of the graft volumes obtained by all biomaterials in the two periods are described in Table 2. The influence of the categorized biomaterial groups according to amounts (weight-grams) used, in the initial and final volumes is described in Table 3. Multiple comparisons among these biomaterial groups were conducted using these data. Concerning the graft volume obtained at V1, no statistical differences were found *p*=0.415.

**Table 2:**
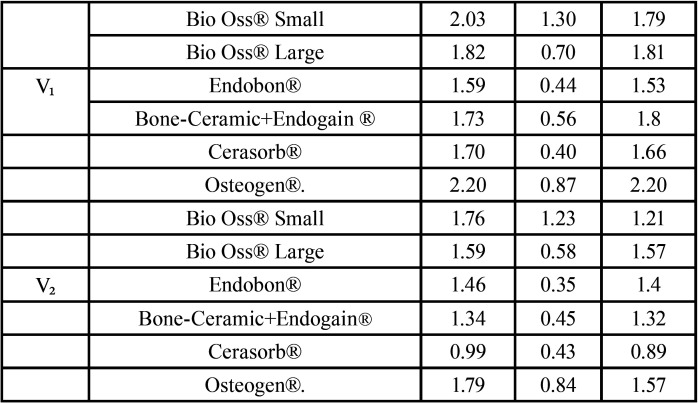
Descriptions of volumes at V1 and V2 in cm3.

**Table 3:**
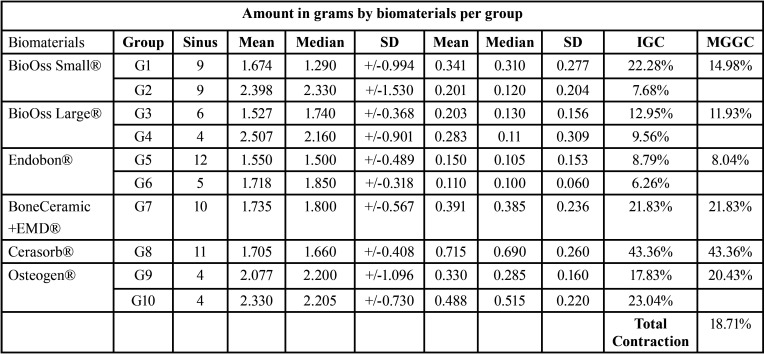
The influence of the categorized biomaterial groups according to amounts (weight-grams) used, in the initial and final volumes. (IGC – Individual group contraction, MGGC – Mean of Graft/Groups Contraction).

The purpose of regression analysis is determining the dependence of a variable on the independent or predictive variable, assuming a linear model. In this way, the degree of linear relationship between the variables is strong or weak, depends on how the group of the points are located around an imaginary line that passes through the set of points.

**Figure 2:**
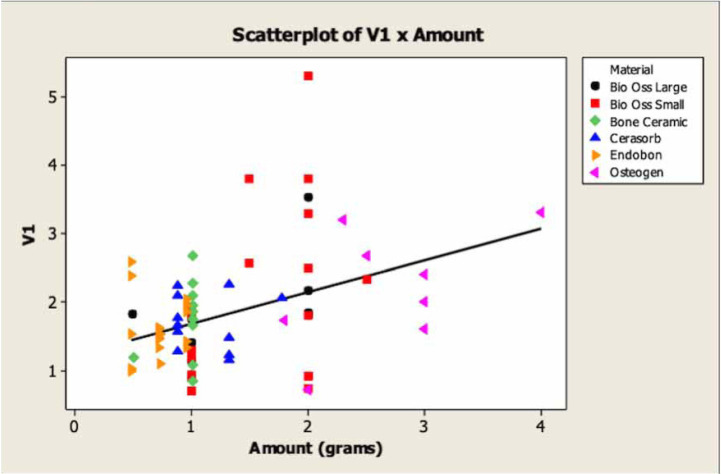
The volume graft obtained at V1 according to the mean amount of the biomaterial (in grams) used.

Figure 2 presents the results of the grafts contractions according to mean amounts (gram weight) of biomaterials used at V1 are shown. It shows that increasing the mean amounts of biomaterials did not increase the grafts volumes at V1. This correlation is weak in the mean amounts of biomaterials with the volumes of the grafts in V1. The biomaterial Osteogen® shows a larger amount in grams used but, did not influence the graft obtained at V1 when compared with the other grafts obtained using other biomaterials.

**Figure 3:**
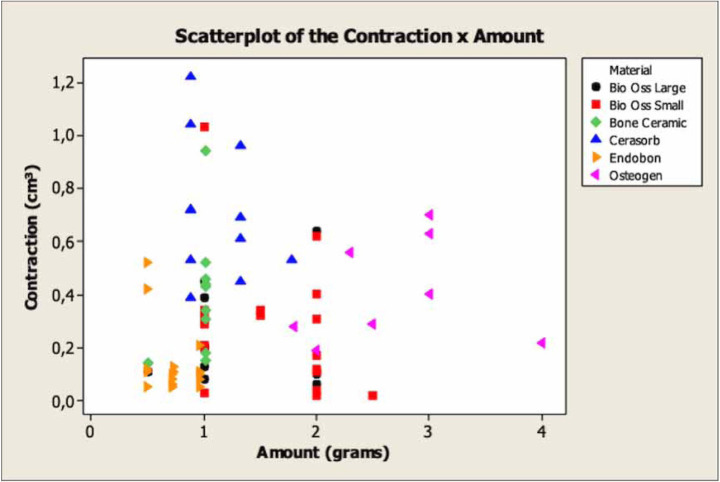
Mean graft volumes at V2 according to the mean amounts of biomaterials in grams used.

Figure 3 shows the linear regression analyses between the mean amounts of biomaterials used and the final mean graft contractions observed at V2. The group of points is distant from the regression line, so in this case it is not formed because the point group is dispersed what represent a weak correlation. The regression analyses at V2 showed that the correlation is very low (close to zero) among the analysed variables, and also show that if the amount of the biomaterials increases the final volume will not increase.

Comparisons of the initial volume (V1) and final volume (V2) according to the biomaterials groups were conducted.

Concerning the final volume obtained at V2 statistically significant differences (*p*=0.00003) can be found only in comparisons of four groups: Cerasorb® G8 x Bio-Oss® Small G2, Cerasorb® G8 x Endobon® G5, Cerasorb® G8 x Endobon® G6 and Cerasorb® G8 x Bio-Oss® Large G3 (Table 4).

**Table 4:**
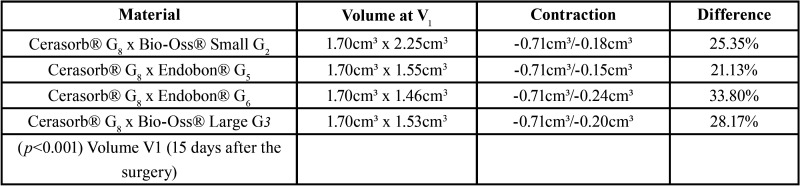
Multiple comparison among groups (Cerasorb® G1 ≥0.89g ≤1.77g, Bio-Oss® small G2 ≥2g ≤ 2.5g, Endobon® G1 ≥0.49g ≤ 0.73g, Endobon® G2 = 0.96g, Bio-Oss® large G1 ≥0.50g ≤1.0g) with statistically significant differences concerning the final volume obtained at V2. (Contractions V1 – V2).

Concerning the distribution of the means obtained at V1 and V2, homogeneous distributions can be found with strong linear correlations for all biomaterials evaluated. A high value for R² (0.887) was found, showing a strong correlation between the variables V1 versus V2. Thus, all biomaterials showed significant contraction between V1 and V2.

## Discussion

The present retrospective cross-sectional cohort observational study has established a correlation of the amount in weight of the xenogeneic hydroxyapatite and alloplastic hydroxyapatite biomaterials with the initial and final volumes of the grafts obtained using these biomaterials in sinus lift. It has also determined the final volume changes of grafts obtained, using CBCT images. The relationships and variables assessed in the present study have still not been identified in the literature.

Among the variables that may influence volumetric changes, it is important to highlight that factors such as the remaining alveolar bone or even total size/volume of the maxillary sinus may also influence the graft volume maintenance. Volume graft reduction should be expected for the use of any biomaterial, particularly at the early stage of graft maturation (21). However, according to Favato *et al.* (13), when evaluating the variable maxillary sinus size/volume with different biomaterials for grafting the maxillary sinus elevation, only determined that the type of biomaterial used in the graft acts as a volumetric alteration factor.

The mean amounts in grams of biomaterials: Bio-Oss® Small (1.58g), Bio-Oss® Large (1.35g), Endobon® (0.72g), BoneCeramic® + Emdogaim® (0.96), Cerasorb® (1.13g) and Osteogen® (2.70g) did not present statistically significant differences. These biomaterials mean amounts were used to establish the correlation between initial and final graft volumes obtained. Therefore, the influence of this variable could be evaluated, and this proposition has not been reported in the literature.

The results of the present study concerning xenogeneic hydroxyapatites Bio-Oss® Small, Bio-Oss® Large and Endobon® have demonstrated that the mean volumes obtained were 2.036cm³, 1.821cm³, 1.599cm3 respectively at V1. For alloplastic hydroxyapatites Bone-Ceramic®, Cerasorb® and Osteogen® were 1.735cm3, 1.75cm3 and 2.204cm3, respectively at V1. The analysis of the correlation of this period shows that if the amount of the biomaterials is increased, the initial volume will not increase. It is suggested that these variations are associated with the structural features particle size and volume weight ratio, from each evaluated biomaterial. Although the limitations of there are no data in the literature to establish a discussion of this variable and just on period of six months in the present study, is important to stresses that the long-term stability of the three-dimensional bone increase is a determining factor for dental implant and aesthetic success according to Shanbhag *et al*. (25).

Thus, regardless of the type of graft biomaterial used, it is clear that it undergoes dimensional changes that may influence the final graft volume over time. In the present study, a final mean contraction of 18.71%, for all biomaterials, was found at the end of the 180-day period. A mean volume reduction of 45% over six months to two years for autogenous bone. Bone substitute biomaterials or mixed grafts may offer greater volumetric stability than autogenous bone alone. Bone substitutes such as demineralized bovine bone or biphasic calcium phosphate, when used alone or in combination with other biomaterials (for example, autogenous bone), present an average 18% to 23% volumetric reduction. Consistent with the literature, (2,6,11,25). this behavior suggests the possible compromise of the stability of the implants installed in these grafts.

Individually analyzed, the mean contractions found for the grafts/biomaterials were: Bio-Oss® Small= 14.98%, Bio-Oss Large= 11.93%, Endobon®= 8.04%, Bone-Ceramic®= 21.83%, Cerasorb®= 43.36% and Osteogen®= 20.43%. A high-volume reduction was observed for Cerasorb® graft, similar to the autogenous graft contraction of up to 40% reduction of the final graft volume. This graft behavior data is consistent with that reported by Browaeys *et al.* (2).

To determine the influence of the amount of biomaterial on the volumetric changes of the grafts, the present study categorized groups by gram intervals and established multiple comparisons. It was found, at final volume V2, that all Cerasorb® groups presented a significant difference in the contractions. These data are very important for the biomaterial choice in sinus lift graft planning.

Concerning the amounts of biomaterials used and the final contractions found with xenogeneic hydroxyapatites in maxillary sinus lift, the similarity of the results to Chackartchi *et al.* (4), Cosso *et al.* (11), Testori *et al*. (15) can be inferred with the data of the present study. In this context, it is difficult to have a more thorough discussion with more specific studies because the scientific information available concerning the influence of the amount of biomaterial used on initial and final graft volumes during maxillary sinus lift has still not been identified in the literature. In contrast to the findings in the literature, which present Cerasorb® (pure β-TCP phase) as a bone substitute comparable to other biomaterials used in maxillary sinus lift (26) the present study shows that Cerasorb® has a higher resorption rate. Analyzing the composition of this biomaterial, it was found to be fully absorbable with no residues of the graft biomaterial. Apparently, the greater amount of the biomaterial was resorbed and replaced by bone, which presented a faster resorption rate than the Bio-Oss® type materials (26). Endobon®, a non-absorbable porous bovine hydroxyapatite with porosity between 45% and 80% (11,28) was the biomaterial that presented less resorption when compared with all the evaluated biomaterials in the present study.

As reported in the literature (3,10,25-30) there is still a deficiency to determine the best osseointegration predictability for installed implants, as well as for aesthetics, concerning the volumetric stability of grafts obtained with different biomaterials after maxillary sinus lift.

Further controlled and longitudinal investigations are needed to verify the impact of the biomaterial amount on the graft volumetric changes using different biomaterials over longer control periods. In this context, studies like the present one take on increasing importance with regard the predictability of osseointegrated implants installed in grafted sinus lift areas.

## Conclusions

Tricalcium phosphate Cerasorb® presented statistically significant contraction were compared to the other biomaterial groups.

The amount of biomaterial used could influence the final graft volume depending on the biomaterial characteristics.
